# Nasopharyngeal carriage of *Streptococcus pneumoniae* serotypes among sick and healthy children in northern India: A case-control study

**DOI:** 10.1016/j.vaccine.2023.09.029

**Published:** 2023-10-20

**Authors:** N. Verma, P. Gupta, A.K. Pandey, S. Awasthi

**Affiliations:** a Department of Pediatrics, King George’s Medical University, Lucknow, Uttar Pradesh, India; b Department of Microbiology, King George’s Medical University, Lucknow, Uttar Pradesh, India

**Keywords:** *Streptococcus pneumonia*, World Health Organization, Community acquired pneumonia, Nasopharyngeal, Vaccine serotypes, Pneumococcal vaccine

## Abstract

**Background:**

*Streptococcus pneumoniae* is leading bacterial cause of community acquired pneumonia and according to World Health Organization, responsible for 14 % death in children. There is effective vaccine available against *Streptococcus pneumoniae*. Hence the primary objective was to isolate *Streptococcus pneumoniae* from nasopharyngeal swabs in children aged 2–59 months with and without community acquired pneumonia and to assess their serotypes.

**Methods:**

This case-control study was conducted in tertiary teaching institutes in northern India. Hospitalized children, aged 2–59 months, with World Health Organization-defined community acquired pneumonia were included as cases. Age matched healthy controls were recruited from immunization clinic. All enrolments were done after written informed parental consent. Nasopharyngeal swabs were taken from both cases and controls, and were cultured on 5 % sheep blood agar with gentamycin plate for growth of *Streptococcus pneumoniae* and incubated in a jar at 37^0^ for 18–24 hrs. Quellung reaction test was used for serotyping.

**Results:**

From March 2017 to December 2022, 2693 children (1910 cases and 783 controls), were recruited. The median age of cases was 7 months and controls 10 months. Almost all the cases had received antibiotics prior to hospitalization. *Streptococcus pneumoniae* positivity in nasopharyngeal swab was 8.1 % in cases, of which 56.8 % were vaccine serotypes and 23.6 % in controls, of which 37.8 % were vaccine serotypes. Adjusted odds ratio of isolating vaccine serotypes among cases as compared to controls was 1.77 (95 % CI, 1.09–2.88).

**Conclusion:**

*Streptococcus pneumoniae* isolation from nasopharyngeal was found to be in lower proportion in cases as compared to control, though colonization with vaccine serotypes was higher in cases as compared to control. Therefore, pneumococcal vaccine coverage must be increased to prevent community acquired pneumonia.

## 1 Introduction

Community acquired pneumonia (CAP) is one of the most common infectious disease of the lower respiratory tract in young children [1], [2], [3]. According to the World Health Organization (WHO), CAP is the main cause of childhood mortality, accounting for 14 % of death in children under 5 years of age [2], [4]. CAP is caused by various bacterial or viral pathogens and less commonly by fungi. Bacterial pneumonia is predominantly caused by *Streptococcus pneumoniae* (SP) in children and this is a common colonizer in the nasopharynx (NP) [2], [5]. The prevalence of SP colonization varies from 7 to 70 % depending on the age and geographic region [6], [7], [8], [9], [10], [11]. In 2018, globally 0.8 million children under age of 5 year died due to SP [12]. Most of these deaths occurred in low and middle-income countries.

The widespread introduction of pneumococcal conjugate vaccine (PCV) has led to reductions in invasive pneumococcal disease and vaccine serotypes (VST) colonization globally [9], [13], [14], [15], [16], [17], [18]. In 2017, India added 13-valent (Prevnar, manufactured by Pfizer) pneumococcal vaccine (PCV13) in the routine immunization program in a phased manner. Since January 2021 the 10-valent (PCV10, Pneumosil, manufactured by Serum Institute of India) [19] is also available in immunization program of the Government of India. PCV is given at 6, 14 and 36 weeks of age. The use of PCV in children was associated with reduced pneumococcal diseases rates. This resulted from the reduced NP colonization of vaccine serotype (VST) of SP in vaccinated children and concomitant reduced transmission [20].

Various studies on pneumococcal colonization have also been conducted among healthy children However in these studies, certain serotypes causing invasion diseases, such as 23F, 19F, 9V, 3 and the prevalence of VST colonization were lower in healthy children than in patients with respiratory diseases [21], [22], [23], [24], [25], [26], [27]. As a result, the prevalence of VST colonization in healthy children may not be reflect the serotypes causing diseases.

Pneumococcal vaccination began in India in 2017 and even at the time of study, coverage was not optimal. SP is the leading bacterial cause of CAP in absence of vaccination against it. SP are carried in nasopharynx and go down the respiratory tract causing pneumonia. Therefore our rationale was to assess NP carriage of SP in healthy children and cases of CAP.

The circulation dynamics of SP are not well known in low and middle income countries since studies are few in these regions. NP carriage rates and their circulating serotypes in the community, particular in children aged <5 years provides useful information for targeting populations for vaccination and future prevention strategies [28]. Therefore, the current study was undertaken with primary objective was to isolate SP from nasopharyngeal swabs in children aged 2–59 months with and without CAP and to assess their serotypes.

## 2 Methods

This cross-sectional multi-centric study was conducted from March 2017 to December 2022 in North Indian states of Uttar Pradesh (Etawah and Lucknow districts) and Bihar (Patna and Darbhanga districs) in children aged between 2 and 59 months after obtaining ethical clearance from the ethics committee of participating institutions and approval of Health Ministry Steering Committee of Indian Council of Medical Research, New Delhi. The development of standard operating procedure, data collection forms, customized, web based data management software were developed and validated.

The data was collected from two tertiary care hospitals from Lucknow, two from Etawah (1 private and 1 tertiary) and one tertiary hospital from Patna and Darbhanga each. Questionnaire was designed to collect information on the status of immunization, socio-demographic, clinical variables, history of presenting symptoms and duration, examination at the time of hospitalization, and whether had the child received medications for the illness, and were the medicines given orally by and/or by injection, prior use of antibiotics and anthropometric measurement. Research team recruited cases from hospitals daily from each study sites based on inclusion criteria. Data was collected from the hospital record files and we obtain the second copy of the x-ray of included patients. Further, chest x-rays were digitalized and interpreted by panel of trained radiologist [29], [30]. PCV13 vaccination data was abstracted from the immunization card of the children. Written informed consent was obtained from the parents before the recruitment of cases and controls.

## 3 Study design

This was a case-control study. Hospitalized cases were recruited from the pediatrics wards. WHO has guidelines for the identification of cases of CAP. According to the guidelines, CAP was defined *“presence of cough and/or difficulty breathing, where the respiratory rate is above age specific range, with or without chest in-drawing is classified as pneumonia. However, children with presence general danger sign namely not able to drink, persistent vomiting, convulsions, lethargic or unconscious, stridor in calm child or severe malnutrition are classified as severe or very severe pneumonia*” [29], [30]. In cases, clinical variables like respiratory rate, heart rate, findings on auscultation of chest were also recorded. Healthy controls were recruited from immunization clinic of pediatric department.

### 3.1 Inclusion criteria for cases

Children aged 2–59 months, with symptoms and signs WHO-defined CAP with lower chest in-drawing and severe CAP, residing in the study district whose parents consent for participation were recruited in the study.

### 3.2 Exclusion criteria for cases

Children whose cough and respiratory symptoms were there for >14 days (to exclude tuberculosis), whose pleural tap/ intercostal drainage had been done prior to hospitalization, who had been admitted within 14 days of discharge from a hospital facility (as they were likely to have nosocomial infections) were excluded in the study.

### 3.3 Inclusion criteria for controls

Healthy children aged 2–59 months, whose parents consented for participation, residing in the study district were recruited as controls.

### 3.4 Exclusion criteria for control

Those with WHO defined CAP or with cough and respiratory symptoms, or diagnosed with any other acute or chronic illness or had received antibiotics in the last 4 weeks were excluded.

### 3.5 Sample collection

NP specimens were collected from children by sterile nylon flocked flexible swabs (Hi Media, India). Immediately swabs were placed in 1.0 ml skimmed milk-tryptone-glucose-glycerol transport (STGG) medium and placed in an ice box as per the WHO consensus methods [20]. Specimens were immediately transported to the microbiology laboratory for culture. All the samples were processed in the laboratory within 1 h.

### 3.6 Laboratory procedure

NP swabs were cultured on 5 % sheep blood agar (Biomerieux, France) and 5 % sheep blood agar with gentamycin (Himedia, India) for growth of SP and incubated in a candle jar at 37 °C for 18–24 h. All pneumococcal isolates were identified by standard microbiological methods [20].

### 3.7 Serotyping of *S. Pneumonaie*

Quellung reaction test was done by using SP isolates of fresh culture. A sterile loopful of the cells of fresh culture were suspended in 50 ul of 0.85 % saline to prepare a suspension. Subsequently, 2 ul of the suspended cells were added on to a glass slide and mixed with 5ul of pooled antiserum and 5 ul methylene blue. These were mixed with a pipette tip. The suspension was covered with the cover slip and incubates at room temperature for 10–15 min. The glass slide was swirled gently while observing for any agglutination reaction until a positive reaction was observed with various pooled antisera. The process was repeated with individual groups with various antisera pools until a positive reaction with the particular serotype specific antisera was observed [20], [31].

#### 3.7.1 Sample size

On the basis of the previous study, the prevalence of the SP in NP of children in Lucknow under 5 years of age among CAP was 34.4 % and in healthy was 41.7 % [28]. For an α = 0.05 level of significance and power 90 % and 3:1 case to control, we required a minimum of 1846 sample in cases and 616 sample in control with total sample size of 2462.

#### 3.7.2 Statistical analysis

Sociodemographic, clinical and laboratory data were entered online in customized software. Sociodemographic variable such as age (in months), gender, and PCV13 status, mother’s education, and immunization status (excluding PCV) of children aged 2–59 months were compared among cases and controls as well as in SP positive cases and controls. Continuous variable were reported as median and interquartile range (IQR) and compared using Mann-Whitney *U* test. The number and percentage was calculated for categorical variables with comparison using chi-square or Fisher’s exact test. A p-value of <0.05 was taken as statistically significant using two-tailed distribution. Statistical package of Social Science (SPSS version 24 Inc., Chicago, United States of America) software was used to perform all statistical analysis. The number and proportion of all vaccine and non-vaccine serotypes among cases and controls were reported individually.

Univariate analysis and multivariate logistic regression analysis was used to assess the association of VST for SP in cases as compared to controls. The unadjusted odds ratio (OR) and adjusted OR with 95 % confidence interval (CI) of isolating VST among cases as compared to controls was calculated. Those variables that had univariate association with p-value <0.1 with SP positive cases vs controls were used in the multivariate logistic regression model.

#### 3.7.3 Results

**Fig. 1:**
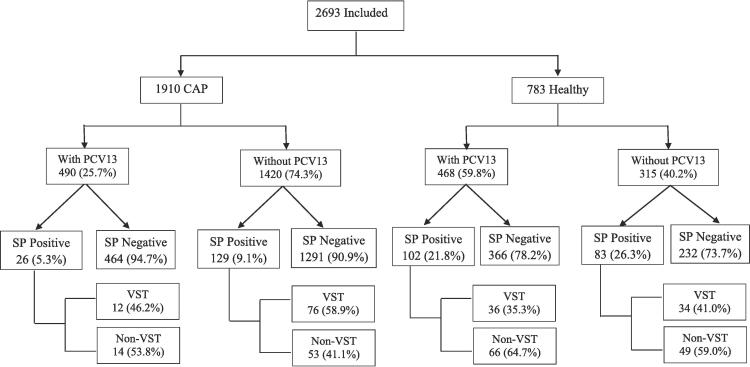
Flow chart of the included children having nasopharyngeal colonization among cases and control. *Abbreviations*: PCV: Pneumococcal conjugate vaccine; SP: *Streptococcus pneumoniae*; CAP: Community acquired pneumonia; VST: Vaccine serotype.

From March 2017 to December 2022, 2693 children were included of which 1910 were cases and 783 controls. Among cases, 25.7 % (490/1910) were PCV vaccinated of which, 5.3 % (26/490) were SP positive; among 74.3 % (1420/1910) children without PCV, 9.1 % (129/1420) were SP positive. Among control, 59.8 % (468/783) were PCV vaccinated of which, 21.8 % (102/468) were SP positive; among 40.2 % (315/783) children without PCV, 26.3 % (83/315) were SP positive. Flow chart of the study given in Fig. 1**.** The median age of children was 10 months (IQR, 5–19 months). Of all recruited cases, 98.3 % (1877/1910) had already taken the prior antibiotics. SP positivity of NP swab was 8.1 % (155/1910) in cases and 23.6 % (185/783) in controls. Comparison of variables among cases and controls are given in Table1.

**Table 1:** Comparison of variables among cases and controls.

Characteristics of variables	Case, N = 1910	Control, N = 783	p-value
Age (in months), median (IQR)	7 (3, 14)	10 (4, 21)	<0.001
Age (2–11 months)	1294 (67.7)	418 (53.4)	<0.001
Gender (female)	628 (32.9)	332 (42.4)	<0.001

**Mother’s education**			
Without formal	690/1891 (36.5)	11/783 (1.4)	<0.001
With formal	1201/1896 (63.6)	772/783 (98.6)	

**Participating sites**			
Lucknow	467 (24.5)	783 (100.0)	
Etawah	609 (31.9)		
Patna	355 (18.6)		
Darbhanga	478 (25.0)		

**Immunization (excluding PCV)**			
Complete for age	1482 (77.6)	776 (99.1)	<0.001
Incomplete/Unimmunized	428 (22.4)	7 (0.9)	

**PCV13 status**			
With PCV	490 (25.7)	468 (59.8)	<0.001
Without PCV	1420 (74.3)	315 (40.2)	

***Streptococcus pneumoniae* in NP swab**			
Yes	155 (8.1)	185 (23.6)	<0.001
No	1755 (91.9)	598 (76.4)	

**Clinical features**			
Fever	1698 (88.9)		
Cough	1886 (98.7)		
Fast breathing	1739 (91.0)		
Difficult breathing	1876 (98.2)		
Wheezing	1149 (60.2)		
Crepitation on auscultation	1569 (82.1)		
Respiratory rate n, mean ± SD	1908, 56.5 ± 12.5		
Heart rate n, mean ± SD	1906, 136.1 ± 18.8		

**Chest x-ray (CXR)**			
With CXR	1708 (89.4)		
Without CXR	202 (10.6)		

**Chest x-ray findings**			
Abnormal	655/1665 (39.3)		
Normal	1010/1665 (60.7)		

*Abbreviations*: PCV: Pneumococcal conjugate vaccine, SP: *Streptococcus pneumoniae*.

**Fig. 2:**
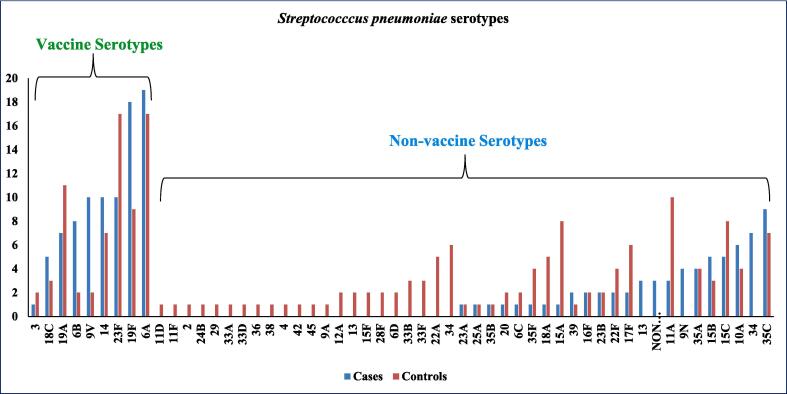
Distribution of *Streptococcus pneumoniae* serotypes in nasopharynx among cases and control.

Distribution of SP serotypes in nasopharynx among cases and controls is shown in Fig. 2. The proportion of serotypes 19F, 6B, 9V, and 15A were significantly different among cases and controls. VST were significantly higher in proportion in cases 56.8 % (88/155) as compared to control 37.8 % (70/185), p < 0.001. VST 6A, 19F, 14, and 23F were predominantly found in cases as well as in controls.

Comparison of variables among cases and controls with nasopharyngeal SP positivity were shown in Table 2. The proportion of all socio-demographic variables was significantly different between cases and controls except in age groups.

**Table 2:** Comparison of variables among cases and controls with nasopharyngeal *Streptococcus pneumoniae positivity*.

Variables	*Streptococcus pneumoniae* positive among cases and controls		
	Case, N = 155	Control, N = 185	p value
Age (2–11 months)	94 (60.6)	101 (54.6)	0.31
Gender (female)	112 (72.3)	100 (54.1)	0.001

**PCV13 status**			
With PCV	26 (16.8)	102 (55.1)	<0.001
Without PCV	129 (83.2)	83 (44.9)	

**Mother’s education**			
Without formal	51 (33.6)	6 (3.2)	<0.001
With formal	101 (66.4)	179 (96.8)	

**Immunization (excluding PCV)**			
Complete for age	118 (76.1)	181 (97.8)	<0.001
Incomplete/Unimmunized	37 (23.9)	4 (2.2)	

*Abbreviations*: PCV: Pneumococcal conjugate vaccine, SP: *Streptococcus pneumoniae,* CAP: Community acquired pneumonia.

Unadjusted odds of isolating VST of SP among cases was 2.16 (95 % CI, 1.40–3.33, p = 0.001), and adjusted odds was 1.77 (95 % CI, 1.09–2.88, p = 0.02), adjusted for immunization (excluding PCV), and PCV vaccination status.

## 4 Discussion

This prospective, multicenter, case-control study was done to assess NP carriage of SP in cases of pneumonia and healthy controls. In our study, while SP positivity in cases were found to be lower in proportion as compared to control, isolation of VSTs of SP were significantly associated with cases. The adjusted odds ratio of isolating VSTs of SP among cases was 1.77 (95 % CI, 1.09–2.88).

In the current study we found 8.1 % carriage rate of SP in NP in cases as compared to 23.6 % in controls. The reported prevalence of SP carriage among healthy children in India ranged from 6.5 to 69.8 % [5], [32], [33], [34], [35]. Neighboring countries of India and some European countries have also reported prevalence of NP carriage of SP in healthy children between 3 and 72.9 % [13], [27], [36], [37], [38], [39], [40], [41], [42], [43]. Cédric Dananché et al. reported 11.86 % carriage of SP in cases from India [28], which is similar to our findings. Since SP is a fastidious bacterium, low isolation of SP from NP of cases could be attributed to high use of antibiotics prior to hospitalization in India. In our study, since almost all the patients had received antibiotics, hence in only 8.1 % of cases SP was isolated from the NP. Other studies have shown isolation rates of 19 % to 40 % which is more than our study [9], [10], [11]. Therefore, serotypes isolated from cases in our study may also be different from the serotypes that could be present in cases that were antibiotics naive.

We found that VST were isolated from 56.8 % of cases and 37.8 % in controls. A study by Cédric Dananch et-al reported 33.3 % to 75.9 % of PCV 13 serotype isolated from cases while in control 40.2 % to 78.1 % [28] which is similar to our study.

PCV13 is effective in reducing the incidence and severity of pneumonia and other lower respiratory infections in children [19]. Therefore, as recommended by the WHO, PCV 13 was introduced in 2017 in India in a phased manner as a part of routine Universal Immunization Program. Studies have reported that high PCV13 coverage is required to interrupt VST pneumococcal transmission and to reduce the burden of VST pneumococcal diseases [44]. Near elimination of VST pneumococcal diseases has predominantly been demonstrated in countries with >90 % vaccine coverage [37]. Two observational studies from USA suggest that statistically significant indirect effects against pneumococcal VST carriage can be achieved even at 58–75 % coverage among children under 5 years of age [45], [46], [47].

Since 2021, PCV10 produced in India is replacing PCV13 which was given by GAVI to the country [16]. PCV 10 contains 1, 5, 6A, 7F, 9V, 14, 19A, 19F, 23F and 6B while PCV 13 has in addition serotypes 3, 4, and 18C. Most of the serotypes in PCV13 are included in PCV10. Therefore, based on the efficacy data, PCV10 and PCV13 would provide protection for the pneumococcal diseases in India. Both vaccines show comparable vaccine efficacies for serotypes contained in the vaccine. Both the vaccine have similar efficacy against SP disease [19].

### 4.1 Strength and limitations

The study has several strengths. We compared the serotype data among cases and controls. Serotyping was done by Quellong method which is the gold standard method for pneumococcal capsular serotyping. Limitation of the study was that we could not collect the NP swab of children prior to their receiving antibiotics. Healthy controls were only recruited in Lucknow. No attempt was made to find the etiology of CAP.

### 4.2 Conclusion

Since cases had received prior antibiotics, SP in NP was found to be in lower proportion among them as compared to control. The colonization with VST of SP was significantly higher in cases as compared to controls. Therefore, pneumococcal vaccine coverage must be increased to prevent community acquired pneumonia.

**Ethical approval**

The study was approved by the Institutional Ethics Committee of King George‘s Medical University, Lucknow vide letter no. 2800 Ethics/R Cell-14 dated 22nd November, 2014.

**Consent to participate**

The caregivers/guardians of children signed the written, informed consent for participation in this study.

**Code availability**

Not applicable.

### CRediT authorship contribution statement

**N. Verma:** Investigation, Data curation, Writing – original draft, Writing – review & editing. **P. Gupta:** Methodology, Investigation, Resources, Writing – original draft, Writing – review & editing, Supervision, Project administration. **A.K. Pandey:** Data curation, Writing – original draft. **S. Awasthi:** Conceptualization, Methodology, Investigation, Resources, Writing – original draft, Writing – review & editing, Supervision, Project administration.

## Declaration of Competing Interest

The authors declare the following financial interests/personal relationships which may be considered as potential competing interests: Prof. Shally Awasthi reports financial support was provided by Bill & Melinda Gates Foundation.

## Data Availability

Data will be made available on request.
